# Color Doppler imaging of cervicocephalic fibromuscular dysplasia

**DOI:** 10.1186/1476-7120-2-7

**Published:** 2004-07-20

**Authors:** Christian Arning, Ulrich Grzyska

**Affiliations:** 1 Department of Neurology, Allgemeines Krankenhaus Hamburg-Wandsbek, Alphonsstr. 14, D-22043 Hamburg, Germany; 2 Department of Neuroradiology, Universitätskrankenhaus Hamburg-Eppendorf, Martinistr. 52, D-20246 Hamburg, Germany

## Abstract

**Background:**

Fibromuscular dysplasia (FMD) is a possible cause of stroke, especially in middle-aged women. However, only few reports are available on ultrasonographic detection and monitoring.

**Methods:**

Among the 15,000 patients who underwent color Doppler imaging (CDI) of the cervicocephalic arteries during the study period, all cases fulfilling ultrasound criteria of FMD were included into the case series. Criteria of FMD were: 1. Segmental string-of-beads pattern, 2. Localization in the distal extracranial part of internal carotid artery (ICA) or vertebral artery (VA), and 3. (optional): Direct and/or indirect criteria of stenosis.

**Results:**

CDI detected FMD in 39 vessels (37 ICA and 2 VA segments) of 21 patients. 16 patients had bilateral manifestation on ICA, one of those also on VA, bilaterally. CDI disclosed 4 symptomatic high-grade ICA stenoses, 3 of them underwent endovascular treatment. 5 patients with moderate symptomatic ICA stenoses got medical treatment. In 6 patients FMD was the most likely cause of headache and in one patient FMD was diagnosed as a cause of vertigo.

**Conclusions:**

CDI may be used for detection of cervicocephalic FMD. Due to the unfavourable localisation of FMD for CDI, the sensitivity of CDI is lower in comparison to angiography. However, high-grade FMD stenoses that require invasive treatment can be recognized on the basis of indirect hemodynamic criteria.

## Background

Fibromuscular dysplasia (FMD) is a non-atheromatous, non-inflammatory arteriopathy of unknown etiology with segmental manifestation on medium-sized arteries in various regions of the body [1]. Manifestation on the renal arteries with the possible consequence of renovascular hypertension is remarkably frequent [2]. The cervico-cephalic arteries, especially the internal carotid artery (ICA) are attacked with an incidence of about 0.6 – 1%, often bilaterally [3]; manifestation also occurs on the vertebral artery (VA) [4]. The disease can occur at any age but is usually diagnosed in middle-aged, predominately female individuals [4].

**Figure 1:**
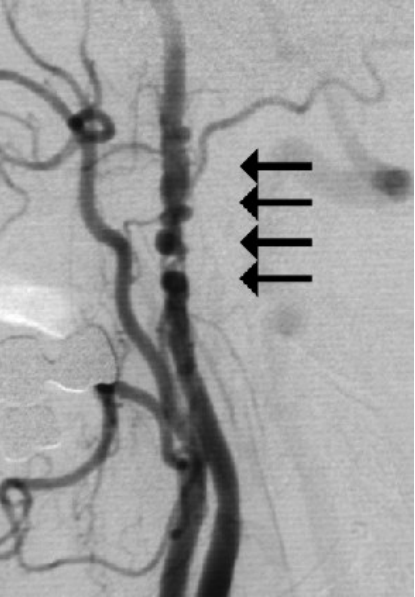
The string-of-beads sign with alternating regions of lumen narrowing and vessel dilatation on angiogram of the ICA (arrows) in a 52-year-old woman sufferning from recurrent transient ischemic attacks.

Angiography reveals in most cases the typical string-of-beads pattern (fig. 1) with alternating regions of lumen narrowing and vessel dilatation over a length of 3 – 5 cm [3]; the proximal section of the ICA is generally not affected, except in a rare FMD subtype characterised by proximal involvement with a web-like membrane [5].

Clinical manifestations of FMD on the ICA are transitoric ischemic attacks or cerebral infarctions [6] as well as unspecific symptoms such as headache and vertigo. In cases of cerebrovascular events, endovascular or surgical treatment is recommended [7-9], therefore detection of FMD is of considerable importance.

## Patients and methods

Among the 15,000 patients who attended the neurosonography department of our clinic during the study period, 21 cases were identified fulfilling ultrasound criteria of FMD (Table 1). The presenting symptoms of the patients are listed in table 2.

**Table 1:** Color Doppler ultrasound criteria of FMD

**1. Morphological criteria:**
Segmental string-of-beads pattern with alternating regions of lumen narrowing and vessel dilatation
**2. Localization:**
Distal extracranial part of ICA (VA).
**3. Hemodynamics **(optional):
Direct and/or indirect criteria of stenosis (in distal extracranial part of ICA / VA).

**Table 2:** Patients and symptoms

**No.**	**Age**	**Male/female**	**Symptoms**
1	52	f	Transient ischemic attack
2	55	f	Bruit
3	55	f	Headache
4	75	f	Bruit
5	61	f	Vertigo
6	53	f	Pulsatile tinnitus
7	63	f	Vertigo
8	65	f	Amaurosis fugax
9	47	f	Amaurosis fugax, vertigo
10	41	f	Minor stroke
11	54	f	Minor stroke
12	52	f	Headache, vertigo
13	57	f	Minor stroke
14	46	f	Vertigo, bruit
15	73	f	Bruit, headache, vertigo
16	55	f	Headache
17	51	f	Headache
18	62	f	Headache
19	42	M	Transient ischemic attack
20	62	f	Transient ischemic attack
21	40	f	Headache

The color Doppler examinations were performed as described by Arning [10] and included the common carotid, external carotid, and internal carotid arteries as well as the vertebral arteries.

CDI was performed with 5 MHz and 7 MHz linear array transducers using one of the following systems: Acuson Sequoia (Siemens AG, Erlangen, Germany), Toshiba Powervision 6000 or Toshiba Aplio (Toshiba Medical Systems Europe, Zoetermeer, Netherlands), or ATL HDI 5000 (Philips Medical Systems, Andover, MA).

## Results

Using the criteria of table 1, FMD was diagnosed in 21 patients (1 male, 20 female). In total, CDI detected FMD in 39 vessels (37 ICA and 2 VA segments). 16 patients had bilateral manifestation on ICA, one of those also on VA, bilaterally. 5 patients had unilateral manifestation on ICA.

**Figure 2:**
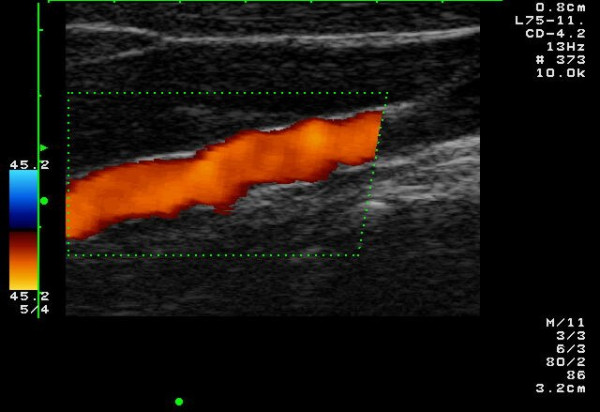
The string-of-beads sign in the color Doppler image in a 51-year-old patient with low-grade stenosing FMD of the ICA. The patient suffered from migraine-like headache.

**Figure 3:**
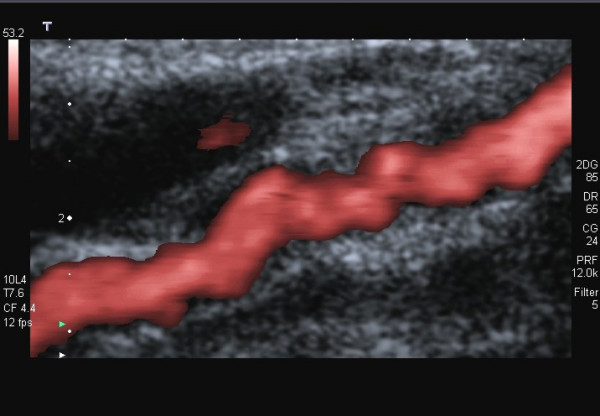
FMD of the ICA in a 53-year-old woman suffering from headache. Power Doppler image of the left ICA shows the string-of-beads pattern.

**Figure 4:**
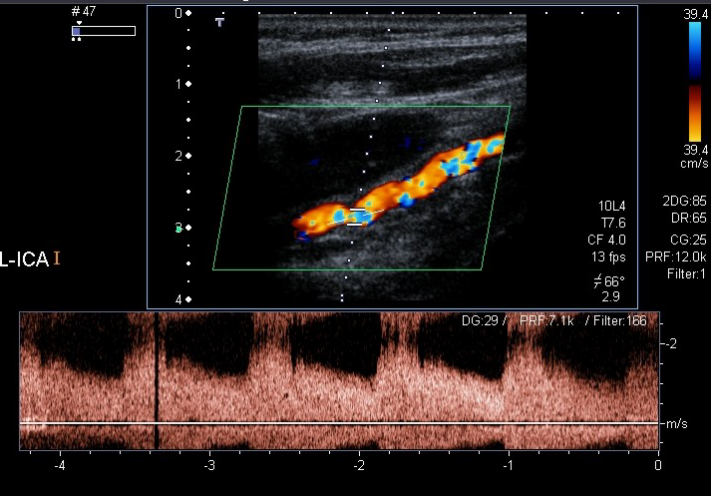
The same case as in fig. 3: Color Doppler and spectral Doppler examination of the left ICA revealing stenoses of about 70%.

**Figure 5:**
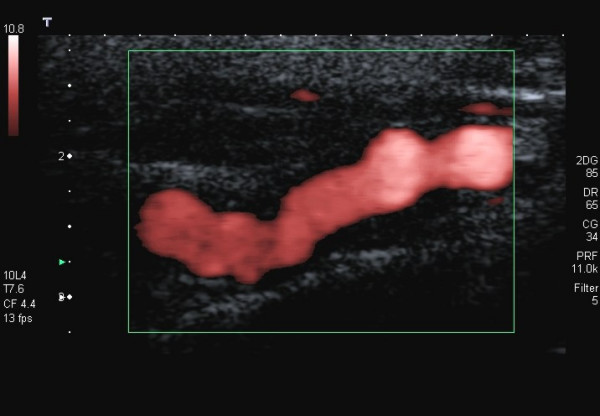
The same case as in fig. 3: Power Doppler image of the right ICA.

**Figure 6:**
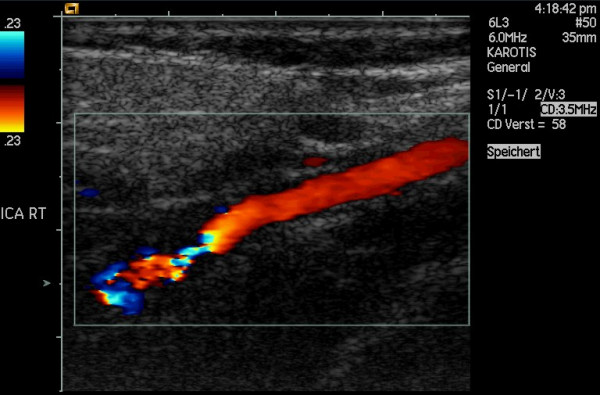
High-grade stenosis of the ICA caused by FMD in a 52-year-old woman sufferning from recurrent transient ischemic attacks. CDI shows the string-of-beads pattern distally to a longer section of normal vessel.

**Figure 7:**
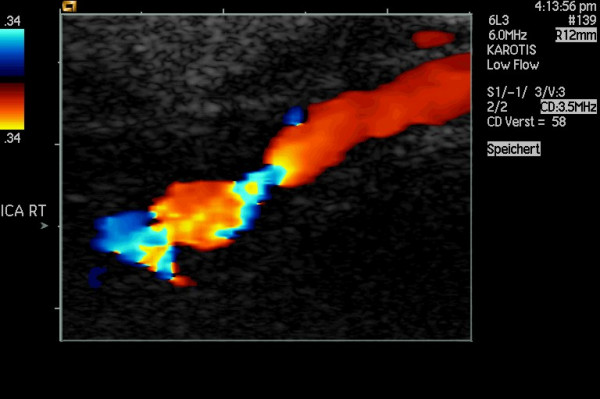
The same case as in fig. 6 (enlarged), showing the string-of-beads pattern distally to a longer section of normal vessel.

**Figure 8:**
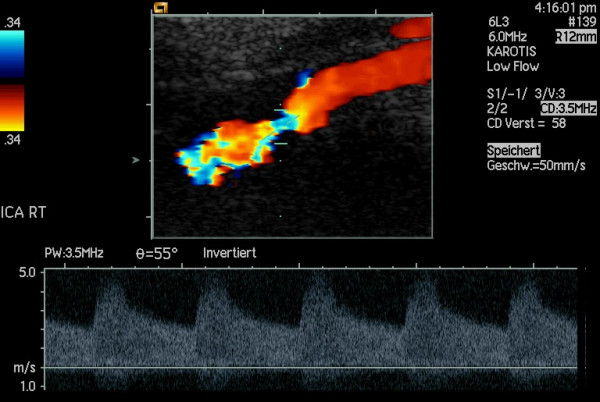
The same case as in fig. 6: Spectral Doppler examinations reveal a high-grade stenosis.

**Figure 9:**
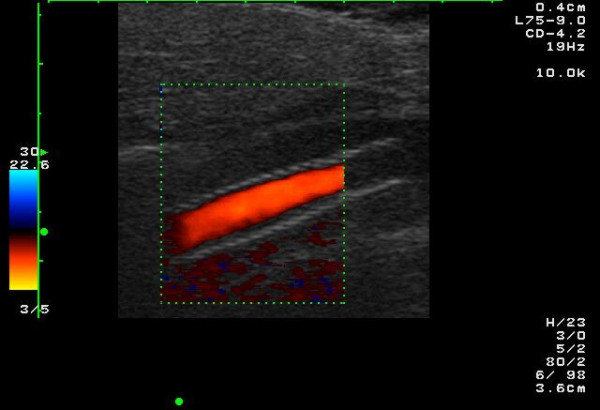
The same case as in fig. 6: Findings after endovascular treatment (stenting).

**Figure 10:**
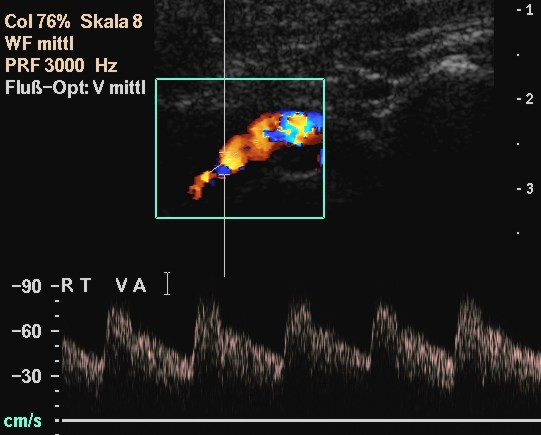
The string-of-beads sign on the VA (C2-C1) in a in a 55-year-old woman with bilateral manifestation of FMD on ICA and VA. The patient suffered from vertigo.

The degree of stenosis was low in 2 patients (Fig. 2) and moderate in the majority of cases (Fig. 3,4,5). 5 patients with moderate symptomatic ICA stenoses got medical treatment. 4 symptomatic high-grade ICA stenoses (Fig. 6,7,8) were detected, 3 of them underwent endovascular treatment (Fig. 9). In 6 patients FMD was the most likely cause of headache and in one patient FMD was diagnosed as the cause of vertigo, involving vertebral artery (fig. 10).

## Discussion

FMD is an uncommon angiopathy with an incidence on the ICA of about 0.6 – 1% [3]. However, the frequency of FMD detection by ultrasound imaging is considerably lower: 0,14% in our case series. Only few reports are available on the detection and monitoring of cervicocephal FMD with ultrasonography [11-14].

Ultrasound criteria of FMD correspond to those of angiography (Fig. 1). CDI reveals the segmental string-of-beads pattern with alternating regions of lumen narrowing and vessel dilatation (Fig. 2,3), distally to a completely normal segment of the vessel (Fig. 6). Dependent on the degree of stenosis, direct (Fig. 8) or indirect hemodynamic criteria may be recognized [14].

In comparison to angiography, the sensitivity of CDI is low: The vascular lesion can only be visualized sonographically when it is located not too far cranially on the ICA [15]. However, high-grade FMD stenoses will be detected on the basis of indirect hemodynamic criteria. To overlook asymptomatic cases of low grade or medium grade stenosing lesions will not have a negative consequence since they do not require any treatment [16].

## Conclusions

CDI allows diagnosis of FMD in numerous cases. Due to the unfavourable localisation of FMD for CDI, the sensitivity of CDI is low in comparison to angiography. However, high-grade FMD stenoses that require invasive treatment can be recognized on the basis of indirect hemodynamic criteria.

## Competing interests

None declared.

## List of abbreviations

CDI Color Doppler Imaging

FMD Fibromuscular Dysplasia

ICA Internal Carotid Artery

VA Vertebral Artery

## References

[B1] Russo CP, Smoker WRK (1996). Nonatheromatous carotid artery disease. Neuroimaging Clinics of North America.

[B2] Slovut DP, Olin JW (2004). Fibromuscular dysplasia. N Engl J Med.

[B3] Sandok BA (1983). Fibromuscular dysplasia of the internal carotid artery. Neurol Clin.

[B4] Mas JL, Bousser MG, Hasboun D, Laplane D (1987). Extracranial vertebral artery dissections: a review of 13 cases. Stroke.

[B5] Morgenlander JC, Goldstein LV (1991). Recurrent transient ischemic attacks and stroke in association with an internal carotid artery web. Stroke.

[B6] Sandmann J, Hojer D, Bewermeyer H, Bamborschke S, Neufang KF (1992). Fibromuscular dysplasia as a cause of cerebral infarct. Nervenarzt.

[B7] Curry TK, Messina LM (2003). Fibromuscular dysplasia: when is intervention warranted?. Semin Vasc Surg.

[B8] Chiche L, Bahnini A, Koskas F, Kieffer E (1997). Occlusive fibromuscular disease of arteries supplying the brain: results of surgical treatment. Ann Vasc Surg.

[B9] Van Damme H, Sakalihasan N, Limet R (1999). Fibromuscular dysplasia of the internal carotid artery. Personal experience with 13 cases and literature review. Acta Chir Belg.

[B10] Arning C (2002). Farbkodierte Duplexsonographie der hirnversorgenden Arterien.

[B11] Edell SL, Huang P (1981). Sonographic demonstration of fibromuscular hyperplasia of the cervical internal carotid artery. Stroke.

[B12] Kliewer MA, Carroll BA (1991). Ultrasound case of the day. Internal carotid artery web (atypical fibromuscular dysplasia). Radiographics.

[B13] Krzanowski M (1997). Fibromuscular dysplasia of the internal carotid artery as a cause of transient cerebral ischemia episodes. Pol Arch Med Wewn.

[B14] Arning C (2001). Nonatherosclerotic disease of the cervical arteries: Role of ultrasonography for diagnosis. VASA.

[B15] Wells RP, Smith RR (1982). Fibromuscular dysplasia of the internal carotid artery: a long term follow-up. Neurosurgery.

[B16] Wesen CA, Elliott BM (1986). Fibromuscular dysplasia of the carotid arteries. Am J Surg.

